# Inducing goat pluripotent stem cells with four transcription factor mRNAs that activate endogenous promoters

**DOI:** 10.1186/s12896-017-0336-7

**Published:** 2017-02-13

**Authors:** Hao Chen, Qisheng Zuo, Yingjie Wang, Jiuzhou Song, Huilin Yang, Yani Zhang, Bichun Li

**Affiliations:** 1 Department of Orthopaedics, The Frist Affiliated Hospital of Soochow University, No. 188 Shizi Street, Suzhou, Jiangsu 215006 People’s Republic of China; 2 Key Laboratory of Animal Breeding Reproduction and Molecular Design for Jiangsu Province, College of Animal Science and Technology, Yangzhou University, 88 South University Ave., Yangzhou, Jiangsu 225009 People’s Republic of China; 3 Animal & Avian Sciences, University of Maryland, Baltimore, MD 20741 USA

**Keywords:** Goat embryonic fibroblasts, Goat iPS cells, Reprogramming, mRNA

## Abstract

**Background:**

Traditional approaches for generating goat pluripotent stem cells (iPSCs) suffer from complexity and low preparation efficiency. Therefore, we tried to derive goat iPSCs with a new method by transfecting exogenous Oct4, Sox2, Klf4 and c-Myc mRNAs into goat embryonic fibroblasts (GEFs), and explore the mechanisms regarding the transcription regulation of the reprogramming factors in goat iPSCs induction.

**Results:**

mRNAs of the four reprogramming factors were transfected into GEFs, and were localized in nucleus with approximately 90% transfection efficiency. After five consecutive transfections, GEFs tended to aggregate by day 10. Clones appeared on day 15–18, and typical embryonic stem cell -like clones formed on day 20. One thousand AKP staining positive clones were achieved in 10^4^ GEFs, with approximately 1.0% induction efficiency. Immunofluorescence staining and qRT-PCR detection of the ESCs markers confirmed the properties of the goat iPSCs. The achieved goat iPSCs could be cultured to 22nd passage, which showed normal karyotype. The goat iPSCs were able to differentiate into embryoid bodies with three germ layers. qRT-PCR and western blot showed activated endogenous pluripotent factors expression in the later phase of mRNA-induced goat iPSCs induction. Epigenetic analysis of the endogenous pluripotent gene Nanog revealed its demethylation status in derived goat iPSCs. Core promoter regions of the four reprogramming factors were determined. Transcription factor binding sites, including Elf-1, AP-2, SP1, C/EBP and MZF1, were identified to be functional in the core promoter regions of these reprogramming genes. Demethylation and deacetylation of the promoters enhanced their transcription activities.

**Conclusions:**

We successfully generated goat iPSCs by transfection of Oct4, Sox2, Klf4 and c-Myc mRNAs into GEFs, which initiated the endogenous reprogramming network and altered the methylation status of pluripotent genes. Core promoter regions and functional transcription binding sites of the four reprogramming genes were identified. Epigenetic regulation was revealed to participate in mRNA induced iPSCs formation. Our study provides a safe and efficient approach for goat. iPSCs generation.

**Electronic supplementary material:**

The online version of this article (doi:10.1186/s12896-017-0336-7) contains supplementary material, which is available to authorized users.

## Background

Embryonic stem cells (ESCs) are pluripotent cells with the capacity for self-renewal [1], differentiation, and rapid proliferation. ESCs were firstly isolated from mouse inner cell mass (ICM) [2], and subsequently isolated from pig [3], monkey [4], rat [5], and other species. It remains difficult to isolate and culture ESCs from goats and other hoofed animals, due to limited knowledge of ESC growth features and culture conditions. Although isolation and cultivation of goat ESCs have been achieved over the past several decades, goat ESC lines have not been established, which largely limited the application of goat ESCs in genetic epidemiology, disease models, neomorphs, and animal species breeding.

The role of genetic engineering of goats has been established in the pharmacy industry, including the production of recombinant proteins, such as antibodies for research and human drugs, or even in advanced clinical trials [6]. In addition, goat is a popular animal model species for research of human diseases. Research in many fields takes the advantage of its high genome homology with humans, which is helpful for optimizing therapeutic protocols and exploring the basic biology [7–9]. The traditional method of establishing an iPS cell line is to transfect inducible factors such as Oct4, Sox2, KIf4, and c-Myc into somatic cells carried by viral vectors. The efficiency of this transfection approach and, the frequency with which all of the inducible factors express simultaneously are very low. Meanwhile, as the viral vectors insert randomly into the somatic cell genome, which may affect the expression quantity of other genes, or cause genetic mutations leading to the expression of proto-oncogenes [10–12]. Although alternative methods using plasmid vectors [13], multiple protein expression [14], small molecules [15], and transposition reduced the integration of exogenous genetic material and improved the security of iPS acquisition [16], these methods suffer from complexity and low preparation efficiency.

mRNA-mediated reprogramming of somatic cells was firstly carried out by Warrens et al. [8] several years ago. They successfully reprogrammed animal somatic cells by using multiple extracorporeal transfections of mRNAs of the reprogramming factors. In principle, this method is in advantage as it avoids the possible insertion of exogenous genes into the somatic cell chromosome. Moreover, mRNA reprogramming may improve guidance efficiency by two orders of magnitude, and double the speed of cell reprogramming. Current efforts to produce iPS using mRNA induction mainly focus on human and mouse, and we are unaware of such work using goat cells.

To produce safe goat iPS cell lines with stable inheritance, in the present study we reprogrammed goat somatic cells using the mRNA of multifunction inducible factors. We verified the feasibility of inducing goat iPS with the mRNA of transcription factors Oct4, Sox2, Klf4, and c-Myc, and analyzed the mechanism of endogenous and exogenous pluripotent genes change during the induction process. We further determined the karyotypes of goat iPS, and explored the transcriptional mechanisms regulating the goat stem cells developmental process.

## Methods

### Preparation of culture medium

Goat embryonic fibroblast (GEFs) culture medium is prepared by adding 10% fetal bovine serum (FBS Gibco-Cat. No.: 10438026), 1% NEAA, 1% L-glutamine and 1% Pen/Strep in high glucose Dulbecco’s Modified Eagle Medium (DMEM Gibco-Cat. No.: 11330057). Goat iPSCs culture medium is prepared by adding 20% KnockOut™ Serum Replacement (KSR Gibco-Cat. No.: 10828028), 1% NEAA, 1% L-glutamine, 0.1 mM EAA, 1% Pen/Strep and 10 ng/mL FGF2 in Knockout DMEM.

### Cell culture

Embryonic fibroblasts were obtained from the fetus of a pregnant goat in Er Ling slaughterhouse in Zhenjiang, Jiangsu. The fibroblasts were isolated, cultured with GEF culture medium, and frozed in liquid nitrogen. The goat iPSCs were cultured in vitro with iPSCs culture medium and fibroblast overnight culture medium (1:1). To obtain the embryoid bodies, the goat iPSCs were cultured in high glucose DMEM with 10% FBS. Fibroblast overnight culture medium was collected by culturing goat and mouse fibroblast cells (1:1) in high glucose DMEM for 24–36 h. The time and the induction efficiency of iPS cell formation was determined by following the protocol from Li et al. [10].

### Vector construction and in vitro transcription

The CDS fragments of Oct4, Sox2, Klf4, and C-Myc were introduced into the MCS region of pCDNA3.0 vectors (NTCC- Cat. No.: 40544312200). We also constructed a pCDNA3.0-EGFP expression vector to assess the transfection efficiency. For maintenance, the resulting vectors (pCDNA3-oct4, pCDNA3-sox2, pCDNA3-klf4, pCDNA3-c-Myc, and pCDNA3-EGFP) were cloned into bacterial cultures, and preserved at −80 °C.

In vitro transcription using the mMESSAGE ®T7 Ultra Kit (Ambion- Cat. No.: AM1340) was performed by following the manufacturer’s protocol. We used the MEGAclear Kit (Ambion, − Cat. No.: AM1908) to purify the synthesized mRNA (purification concentration range: 100–500 ng/μL). DAPI was purchased from Invitrogen.

### mRNA transfection and culture of reprogramming cells

GEFs were incubated in 24-well plates. When the density reached 50 ~ 60%, transfection was performed. 1 ~ 2 h before transfection, 400 μL opti-MEM medium was added to each well. Transfection cocktails were evenly mixed in sterilized PCR tubes by combining 0.2 μg mRNA for each transcription factor with EGFP (1 μg total mRNA), 1 μL lipo-2000 and 100 μL opti-MEM. The mixtures were kept at room temperature for 20 min, and subsequently added to the 24-well plates. All groups were cultured at 37 °C with 5% CO2 for 6 h, and the medium was subsequently replaced with GEFs culture medium. We performed four additional transfections on each well with the interval of 24 h. Cells were passaged on day 6 using goat iPSCs culture medium. Cells were passaged on days 10, 15, and 19 using trypsin (Gibco- Cat. No.: 25300054). On day 24, iPSCs were trypsinized, cultured, and identified.

### Quantitative real-time PCR (qRT-PCR)

After digestion with 0.25% trypsin, GEF cells from post-transfection day 1, 6, 9, 12, 15, 18, and 21 were collected separately for RNA extraction and cDNA synthesis *via* reverse transcription. We performed qRT-PCR using SYBR fluorescent reagent with a 7500 System florescence quantitative instrument (Thermo- Cat. No.: 7500 fast) by following the PCR kit instructions (Thermo- Cat. No.: 11731023). Data were analyzed by 2^−ΔΔCt^ relative quantification in the Microsoft Excel software package. The primer sequences for qRT-PCR were shown in Additional file 1: Table S1.

### Western blot

The whole lysate of GEFs from post-transfection day 1, 6, 9, 12, 15, 18, and 21 was extracted by following the protocol recommended by the protein extraction kit manufacturer. Western blots were performed by following the methods reported [17]. The detail antibody information were provided as below: Oct 4 (Abcam- Cat. No.: ab19857, dilution ratio 1:1000), Sox 2 (Abcam- Cat. No.:ab97959, dilution ratio 1:1000), Klf 4 (Abcam- Cat. No.: ab72543, dilution ratio 1:1000), C-Myc (BD Biosciences- Cat. No.: 551101, dilution ratio 1:1000), Nanog (Abcam- Cat. No.: ab21624, dilution ratio 1:1000), β-actin (Abcam- Cat. No.: ab8226, dilution ratio 1:1000), goat anti-mouse IgM [FITC] labeled (Abcam - Cat. No.: ab8227, dilution ratio 1:1000).

### AKP staining and indirect immunofluorescence

Goat iPS cells were stained according to the AKP staining kit instructions (SiDanSai- Cat. No.: 1101–050). We washed the cultured cells 24 h and 21 d post-transfection with PBS for 2–3 times. We subsequently performed indirect immunofluorescence by following the method of Zhang et al. [11]. The dilution ratio of anti-rabbit antibody was 1:1000, and the dilution ratio of FITC-labeled goat anti-rabbit secondary antibody was 1:1000. We added DAPI at a ratio of 1:100, and performed nuclear staining for 10 min. We observed and photographed the cells using a fluorescence microscope (Olympus- Cat. No.: IX51). The detail antibody information were provided as below: OCT4 (Abcam- Cat. No.: ab19857, dilution ratio 1:500), SOX2 (Abcam- Cat. No.:ab97959, dilution ratio 1:500), KLF4 (Abcam- Cat. No.: ab72543, dilution ratio 1:500), C-MYC (BD Biosciences- Cat. No.: 551101, dilution ratio 1:500), CDX2 (BD Biosciences- Cat. No.: 560171, dilution ratio 1:500), REX (Abcam- Cat. No.: ab50828, dilution ratio 1:500), SSEA-1(BD Biosciences- Cat. No.: 561585, dilution ratio 1:500), TRA-1-60 (BD Biosciences- Cat. No.: 560884, dilution ratio 1:500), TRA-1-81 (BD Biosciences- Cat. No.: 560072, dilution ratio 1:500).

### Differentiation into targeted cells types

After culturing goat iPS cells for 4–7 d in high glucose DMEM containing 10% FBS, we observed embryoid bodies. We transferred them into gelatin-coated flasks (Sigma- Cat. No.: 9000-70-8). Different cell morphologies were observed after few days culture, and cells were identified by immunofluorescence. The dilution ratio for SOX17 (endoderm) (R & D), Smooth Muscle Actin (SMA; mesoderm) (Santa Cruz), and (endoderm) (R & D), Smooth Muscle Actin (SMA; mesoderm) (ies were 1:100. The dilution ratio of FITC-labeled goat anti-rabbit secondary antibody was 1:1000. SMA (Abbiotec- Cat. No.: 252037, dilution ratio 1:500), Sox17 (BD Biosciences- Cat. No.: 561590, dilution ratio 1:500), Tuj-1(MyBioSource- Cat. No.: MBS530431, dilution ratio 1:500).

### Bisulfite genomic sequencing

We extracted the genomic DNA from non-transfected and goat iPSCs. We used a CpGenome Modification Kit (Millipore-Cat. No.: S7820) to perform the bisulfite treatment according to the manufacturer’s protocol. The PCR-amplified products were ligated into T-vector and cloned into bacteria. Ten clonal colonies were selected and sequenced.

### G-banding karyotype analysis

When GEFs and goat iPSCs were in the logarithmic growth phase, we passaged these cells into media containing colchicine (final concentration: 0.1 μg/mL) and cultured for 2–3 h. After digestion, cell media was removed. Following the method reported in Zhang Jingnan et al. [18], we prepared Arabian horse chromosome G-banding karyotype, and detected goat iPS cell karyotypes. As G-banding appeared, we digested the cells with pre-warmed 0.25% trypsin for 15 ~ 20 s, and washed the cells with saline. Then, the products were stained with 10% Giemsa for 10 min. The slides were subsequently rinsed, air-dried, observed, and photographed under an optical microscope. Colchicine (Cat. No :U3385), Triton X-100 (Cat. No : 21123), and Giemsa (Cat. No : 48900) were purchased from Sigma.

### Deletions cloning and epigenetic induction of goat Sox2, c-Myc, and Oct4 gene promoters

The key transcription factor promoters were amplified and inserted into dual-luciferase vectors for dual-luciferase activity assays. Meanwhile we performed site-specific mutagenesis on transcription factor binding sites in core promoter regions. 5-Azadc and TSA were used for epigenetic modifications.

Taking pMD19-Sox2pro1841, pMD19-c-Mycpro1976 and pMD19-Oct41966 as amplification template, specific primers amplified a different deletion of the Sox2, c-Myc and Oct4 promoters. Reactions were carried out using the methylation inhibitor 5-Azadc (5-aza-2′- deoxycytidine) at 1, 5, 10, and 15 μM, and histone deacetylase inhibitor trichostatin A (TSA) at 1, 2, 4, and 6 mM VPA (Valproic acid), and nuclear factor of activated T cells (NFAT1) at 0.1, 0.5, 1, and 1.5 μM for induction. After allowing the reactions to proceed for 24 h, we performed dual-luciferase reporter assays by following the standard protocol included with the Promega double fluorescence detection kit (Promega-Cat. No.: E1910).

## Results

### Generation of mRNA induced goat iPSCs

**Fig. 1:**
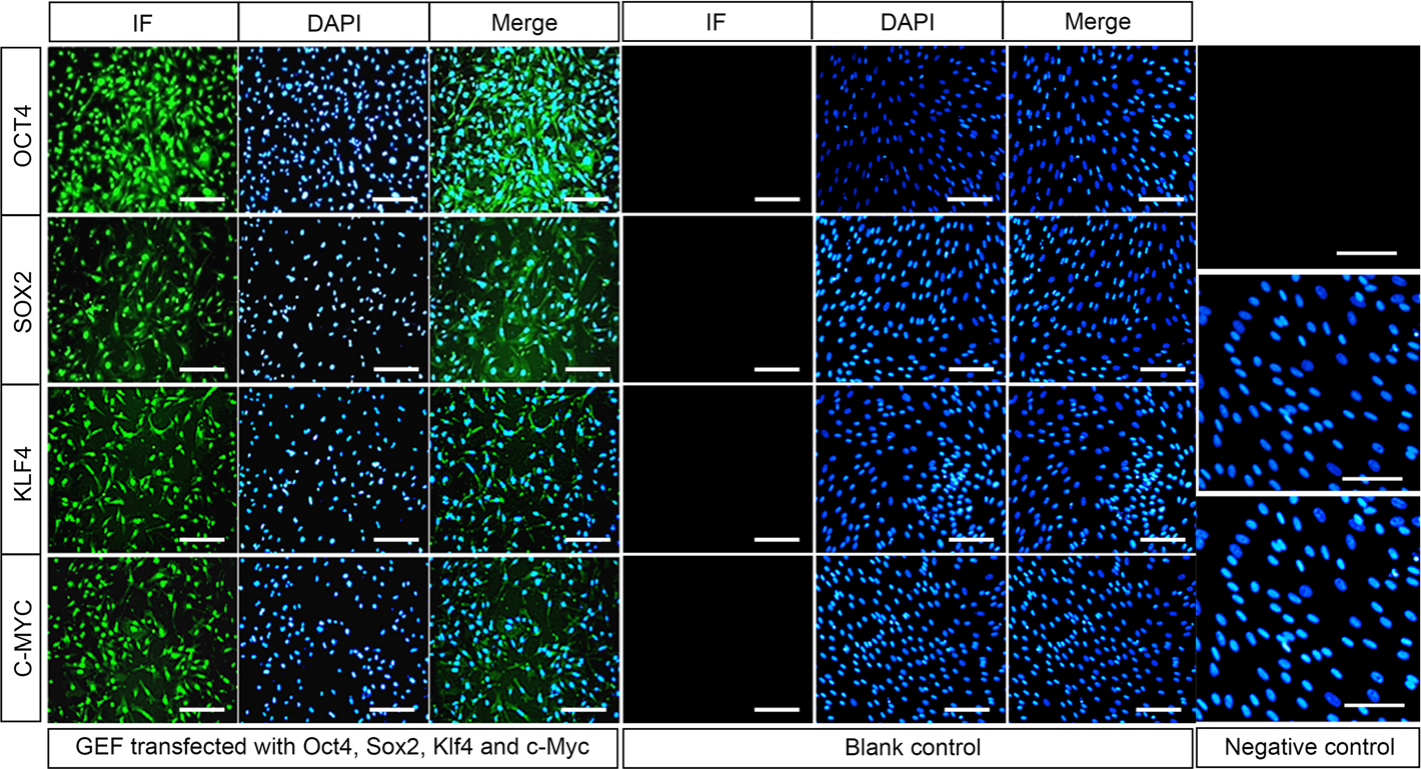
Transfection and localization of Oct4, Sox2, Klf4 and c-Myc in GEFs. Indirect immunofluorescence staining was used to detect the transfection of mRNA from the four reprogramming factors Oct4, Sox2, Klf4 and c-Myc. After five consecutive transfections, expression of the four reprogramming factors were observed in GEFs, and localized in the nucleus. GEFs without transfection were used as the blank control. Immunofluorescence staining without primary antibodies was employed as the negative control. Neither blank control or negative control showed positive staining. Scale bar: 50 μm

**Fig. 2:**
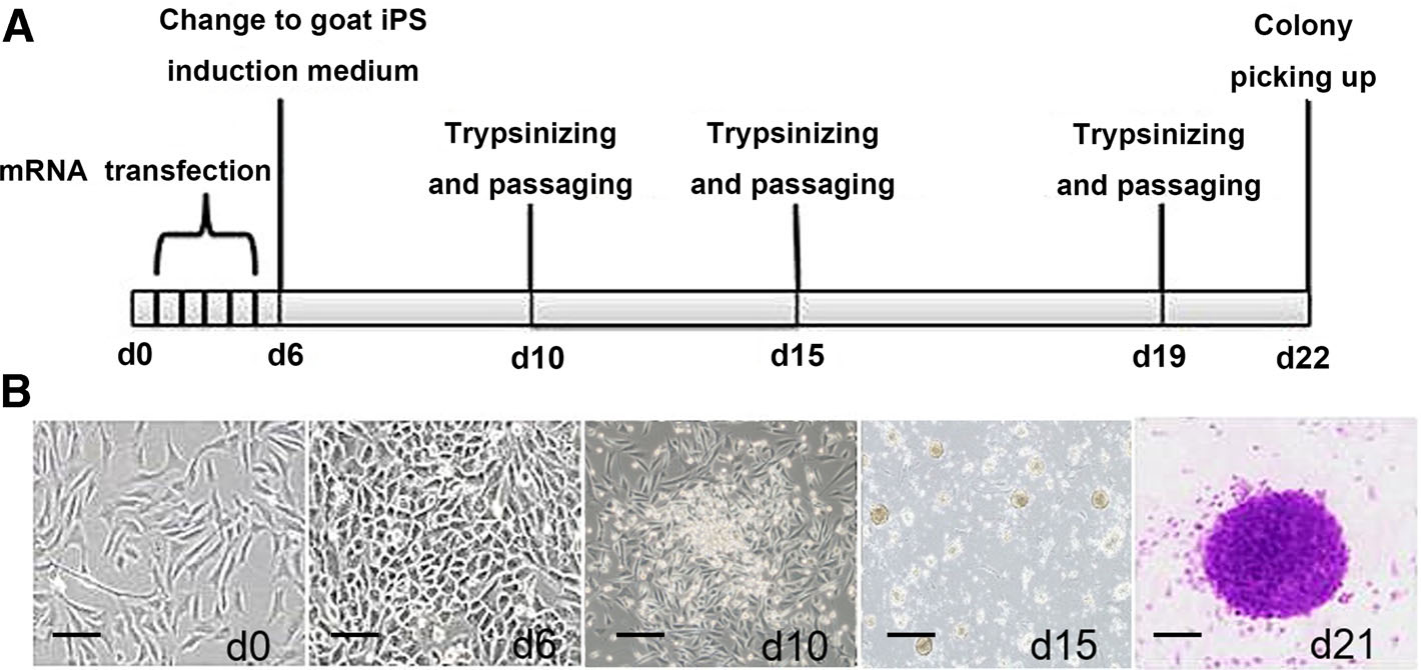
Dynamic changes of the GEFs morphology during goat iPSCs induction. **a** The schematic diagram of mRNA induced goat iPSCs induction process. **b** GEFs morphology continuously changed during iPSCs induction. GEFs without transfection were shown in the shape of spindle. Six days after mRNA transfection, the culturing medium was changed to iPSCs induction medium, and the GEFs became round. On day 10, the cells exhibited aggregation growth feature. On day 15, small clone-like cells appeared. On day 21, goat ESC-like clones with clear cell borders were observed, which were positively stained with AKP. Scale bar: 50 μm

To evaluate the transfection efficiency and localize the four transcription factors in GEFs, we performed indirect immunofluorescence assay. The results showed the Oct4, Sox2, Klf4, and c-Myc mRNAs had been successfully transfected into the GEFs with the efficiency more than 90%, and all the four reprogramming factors were localized in the nucleus (Fig. 1). Six days after mRNA transfection, the GEFs morphology started to change from spindle to round, and the cells were observed to proliferate rapidly (Fig. 2). The cells exhibited aggregation growth on day 10. Small clone-like cells could be seen on day 15. Subsequently, we observed large, round ESC-like cells with clear cell bounders on day 19 and typical flat and dense goat ES-like clones on day 20 (Fig. 2). Goat iPS cells were presented as round and tight colonies. They have high cytoplasmic ratio and obvious endoblasts, which were similar to the morphology of mouse and sheep ESCs (Fig. 2b). Totally, we obtained about 1000 AKP positive clones from the initial 10^4^ GEFs, indicating the induction rate was approximately 1%. The results above imply that mRNAs of the reprogramming factors can be transfected into GEFs and exert function in the nucleus, and induce GEFs into iPSCs.

### Characterization of derived goat iPS cell line

**Fig. 3:**
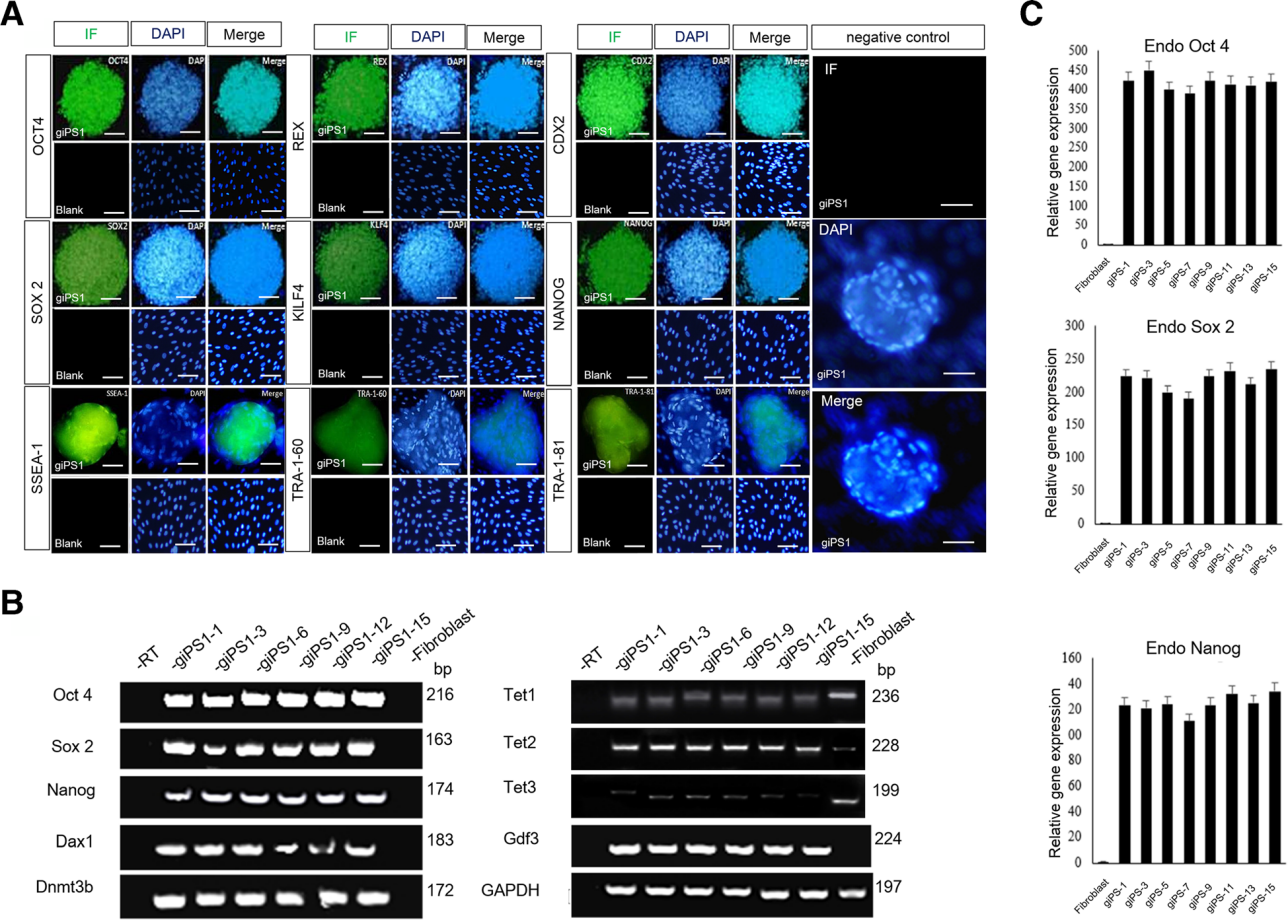
Expression profiles of the derived goat iPSCs. **a** the properties of goat iPS1 cell line were evaluated with ESCs markers, including OCT4, SOX2, KLF 4, NANOG, CDX2, REX, SSEA-1, TRA-1-60 and TRA-1-81. All these markers were positively stained in the goat iPS cell line. GEFs without transfections were used as the blank control, which did not express ESCs markers. Immunofluorescence staining of goat iPS1 cells without primary antibodies was used as the negative control. Scale bar: 50 μm. **b** qRT-PCR analysis confirmed that the goat iPS1 cell line express a variety of ESCs marker genes, including Oct4, Sox2, Nanog, Dax1 and Gdf3. The expression of DNA methyltranferase Dnmt3b and DNA dymethyltransferase TET1, 2, 3 were also detected with qRT-PCR. **c** Quantitative evaluation of Oct4, Sox2, and Nanog expression in different passages of goat iPS1 cell line. Up: Oct4, Middle: Sox2, Down: Nanog

With the approach mentioned above, we established a goat iPS cell line (goat iPS1) that stably passaged for over 22 generations (>80 d). The doubling time of the iPS cell line was about 21.5 h. The cloning efficiency for the F2 and F3 generations were 62.8 ± 0.17% and 36.8 ± 0.21%, respectively. The clones formed in goat iPS1 cell line could be positively stained with AKP (Fig. 2b). Immunofluorescence staining revealed that the goat iPS1 cell line express goat ESCs markers, including OCT4, SOX2, KLF4, C-MYC, NANOG, REX1, SSEA-1, TRA-1-60 and TRA-1-81 (Fig. 3a). The expression of ESCs markers of goat iPSCs was further confirmed by qRT-PCR, and the results showed that ESCs marker genes Oct4, Sox2, Nanog, Dax1 and Gdf3 were highly expressed in goat iPSCs (Fig. 3b). Among all the markers detected, the expression of NANOG and REX-1 implied the activation of endogenous reprogramming network. In addition, qRT-PCR also revealed that goat iPS1 maintained a relatively stable expression of Oct4, Sox2, and Nanog in passages (Fig. 3b). Meanwhile, the expression of DNA methyltranferase Dnmt3b and DNA dymethyltransferase TET1, 2, 3 was also detected with qRT-PCR. Dnmt3b was expressed in goat iPS1 cell line, but not in GEFs, whereas, TET1 and TET3 were nearly not detected in goat iPS1 cell line, confirming the initiation of reprogramming process in goat iPS cell line (Fig. 3b). The results above indicate the ESC-like properties of the goat iPS1 cell line we derived.

**Fig. 4:**
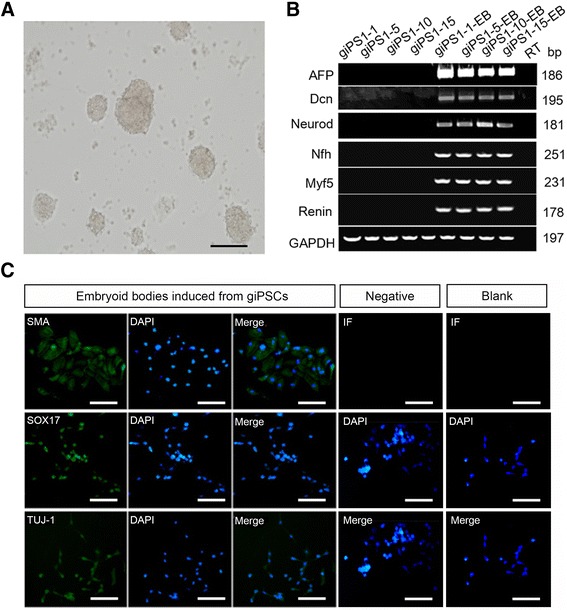
Goat iPSCs is able to differentiate into three germ layer cells. **a** Morphology of embryoid bodies derived from the goat iPS1 cell line. Scale bar: 50 μm. **b** qRT-PCR evaluation of the three germ layer marker genes expression was performed on the embryoid bodies derived from different generations of the goat iPS1 cell line. AFP and DCN (endoderm), NeuroD and NFH (ectoderm), Myf5 and Renin (mesoderm). The emboyid bodies derived express these marker genes. **c** Immunocytochemistry staining of the three germ layers markers, smooth muscle actin (SMA), Sox17, and βIII-tubulin (TUJ-1), were conducted, showing positive staining of the emboyid bodies induced from the goat iPSCs. iPSCs without induction were used as the blank control, which did not express the three markers. Immunofluorescence staining of the induced embyoid cells without primary antibodies was used as the negative control. *Scale bar*: 50 μm

As the ESCs are capable to differentiate into any of the three germ layers*,* we, therefore, assessed this ability on goat iPS cell line we derived. The goat iPS1 cells were cultured in high glucose DMEM medium with 10% FBS for 4 to 7 days to form the embryoid bodies (Fig. 4a). The embryoid bodies were then detected with the markers represent for three germ layers. qRT-PCR analysis showed that the goat iPSCs had differentiated into cells with the characteristics of all the three germ layers, as they express AFP (endoderm), DCN (endoderm), NeuroD (ectoderm), NFH (ectoderm), Myf5 (mesoderm) and Renin (mesoderm) (Fig. 4b). To further validate this findings with immunofluorescence staining, the obtained embryoid bodies were trypsinized and cultured in flasks coated with gelatin, and cultured for several days. Immunofluorescence results showed that the cells were positively stained with all the three germ layers markers including SMA, SOX17 and TUJ-1. The results above demonstrated that the goat iPSCs we induced share the property of pluripotency as that in the ESCs (Fig. 4c).

### Reprogramming mechanism in mRNA induced goat iPSCs

**Fig. 5:**
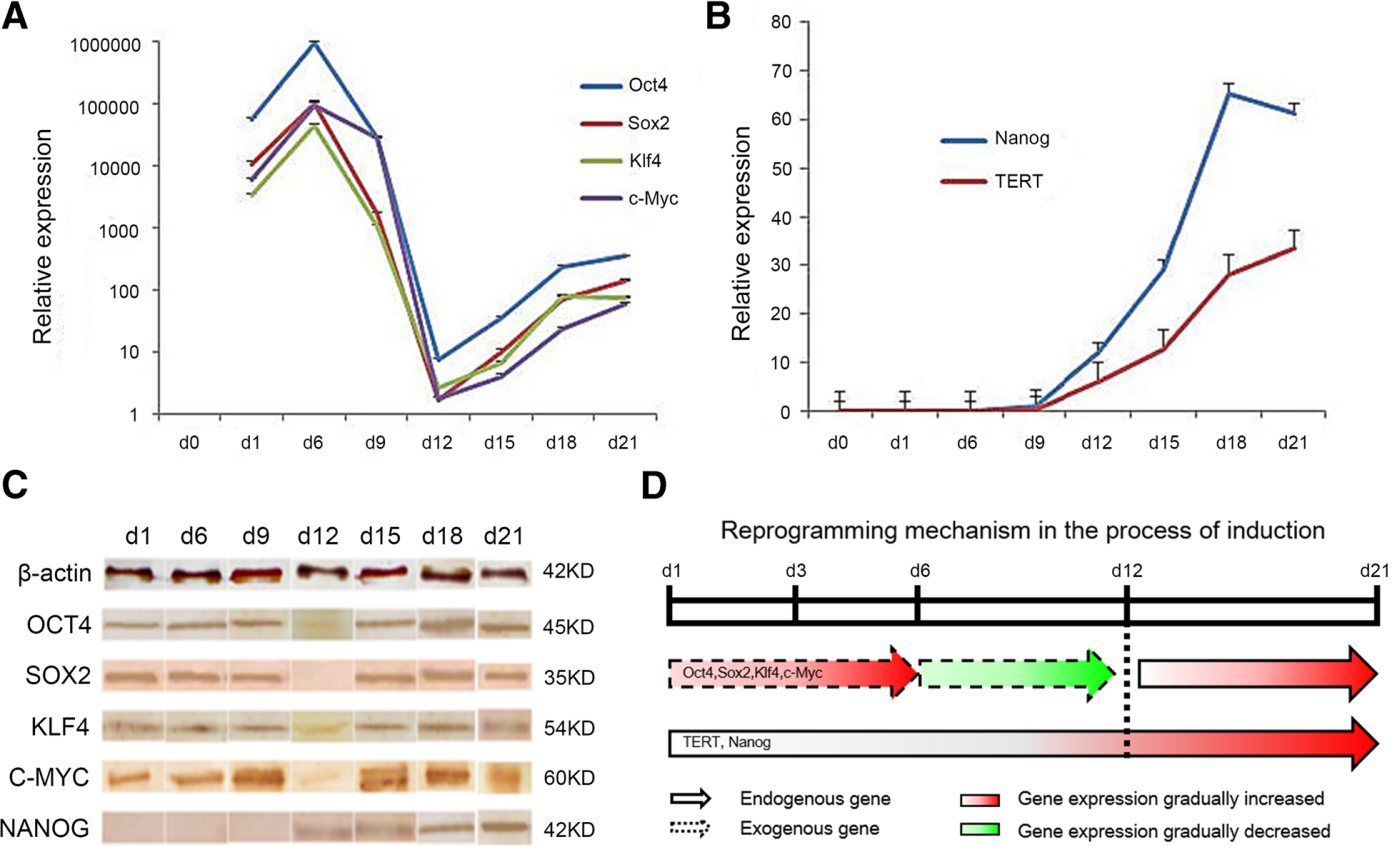
Reprogramming mechanism in the process of mRNA induction. **a** The expression of Oct4, Sox2, Klf4, and c-Myc were evaluated by qRT-PCR. Their expression reached the maximum 6 days after transfection and then decreased to the minimum on day 12. Then, the expression of these reprogramming factors increased gradually afterwards. **b** Expression of the endogenous pluripotent genes Nanog and TERT began to increase on day 12. **c** Western blot analysis of the OCT4, SOX2, KlLF4, C-MYC and NANOG during mRNA-induction process. d1, d6, d9, d12, d15, d18, d21 represent the day after exogenous mRNA transfection into GEFs. **d** Schematic diagram of the endogenous and exogenous expression pattern of the reprogramming markers during mRNA-induced GEFs reprogramming process

To understand the induction process of goat iPSCs, we evaluated the expression of the pluripotent markers with qRT-PCR and western blots. It showed that the expression of Oct4, Sox2, Klf4, and c-Myc mRNAs reached its maximum on day six after transfection and then decreased gradually (Fig. 5a). On day 12, the four genes expression was almost disappeared. After that, their expression, along with endogenous pluripotent genes Nanog and TERT, began to increase (Fig. 5a and b). Western blots analysis of OCT4, SOX2, KLF4, C-MYC and NANOG further confirmed their expression pattern in protein level (Fig. 5c). The results indicate that exogenous Oct4, Sox2, Klf4, and c-Myc gradually decreased to its minimum by day 12, and the endogenous expression of the pluripotent factors was subsequently initiated (Fig. 5d). These results suggest that the endogenous reprogramming network could be activated by exogenous mRNAs in the reprogramming process.

### Epigenetic and genetic alternations in mRNA induced goat iPSCs

**Fig. 6:**
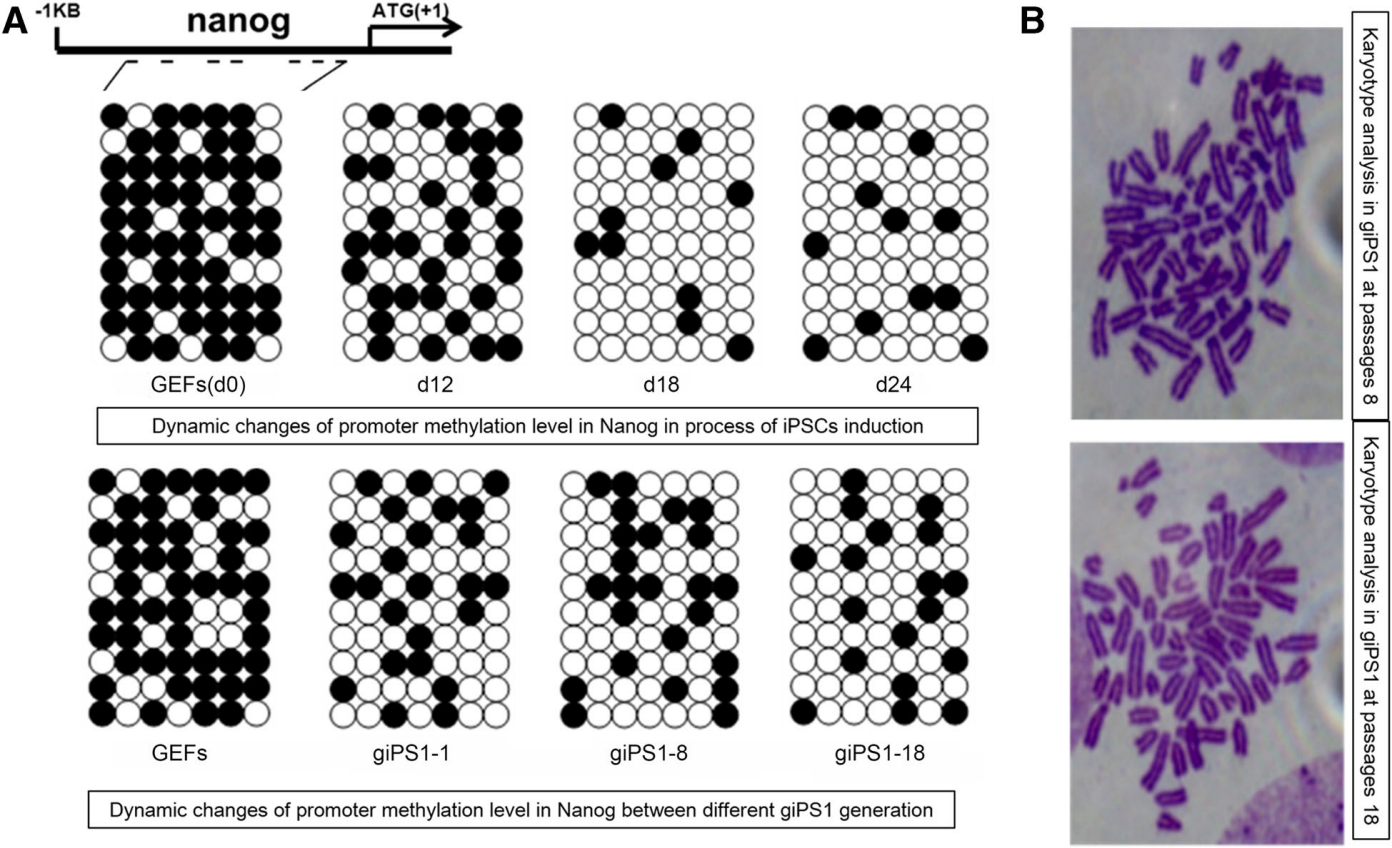
Epigenetic and genetic changes of goat iPSCs. **a** Bisulfite genomic sequencing of the key pluripotent gene Nanog promoter area showed reduced methylation level during goat iPSCs generation, and the low methylation status was maintained in goat iPS1 passages from 1 to 18. The open and closed circles represent the unmethylated and methylated CpGs, respectively. GEFs without transfection was used as the negative control **b** Karyotype analysis of the goat iPS1 cell line at passages 8 and 18 showed normal 58 XY karyotypes

As epigenetic regulation has been identified to possibly regulate iPSCs formation, we analyzed the dynamic change of the epigenetic status by performing bisulfite genomic sequencing of the promoter in the endogenous gene Nanog. Our results demonstrated that the CpG island methylation level in the Nanog promoter region decreased gradually alone with the formation of goat iPSCs, and the unmethylated status was observed to maintain in passages of the goat iPS cell line (Fig. 6a). Meanwhile, the goat iPSCs at generation 10 and 20 showed normal karyotype with 58 autosomes and two sex chromosomes during passage (Fig. 6b). Combine with previous qRT-PCR and western blot results (Fig. 5), these results strongly indicate that the endogenous Nanog gene promoter was reactivated during goat iPSCs generation.

### Initiation mechanism of the reprogramming gene promoters in goat iPSCs induction

**Fig. 7:**
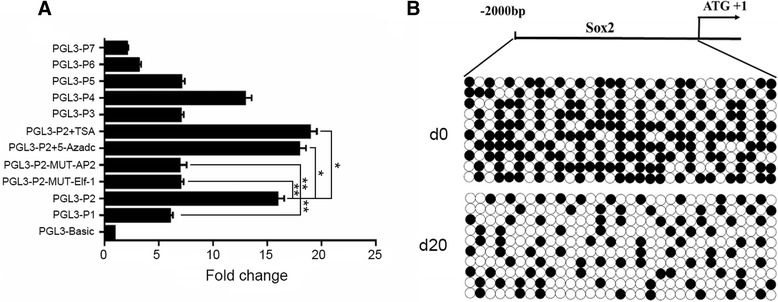
Analysis of goat Sox2 promoter. **a** Goat Sox2 promoter activity was assessed by dual luciferase reporter assay of different promoter deletions, and the PGL3-P2 deletion showed the highest activity among all the deletions we colned. Loss the function of AP-2 and Elf-1 transcription factor binding sites significantly reduced the promoter activity. Treatment with TSA and 5-Azadc significantly improved the activity of PGL3-P2. (* represents for *P* value <0.05, and ** represents for *P* value <0.01) **b** Bisulfite genomic sequencing of the Sox2 promoter showed 31 modified CpG sites, among which, the methylated sites were significantly reduced in derived goat iPSCs

To understand the mechanisms of reprogramming factor promoters in goat iPSCs induction, we analyzed the promoter regions of the four key genes. Different deletions of the Sox2, c-Myc, Oct4 and Klf4 promoters were cloned and inserted into PGL-3 vector, for which the activity of each deletion was evaluated by dual luciferase assay. The results showed the core promoter of Sox2 gene was located in the region of −1298 ~ + 49 bp (PGL3-P2) (Fig. 7a). Meanwhile, the transcription binding sites of the Sox2 core promoter were predicted, and the corresponding binding site-directed mutation vectors were constructed. Dual luciferase assays demonstrated that, the key transcription factor binding sites located in Sox2 core promoter region were Elf-1 and AP-2 (Fig. 7a). Furthermore, to explore the epigenetic regulation of the reprogramming factors, 10 μM 5-Azadc and 1 μM of TSA were used to demethylate and deacetylate the Sox2 core promoter. The results indicate that demethylation and deacetylation of the Sox2 core promoter could significantly enhance the activity (Fig. 7a). The methylation status of the Sox2 promoter was further confirmed by bisulfite genomic sequencing, and it clearly showed that the promoter methylation was declined significantly after 20 days induction (Fig. 7b). Besides Sox2, we also explored the core regions and epigenetic status of c-Myc, Oct4 and Klf4 promoters, and all of these factors exhibited similar modifications during goat iPSCs induction (data not shown).

## Discussion

The present study demonstrated that transfection of multiple transcription factor mRNAs is able to reprogram goat somatic cells into iPS cells. Compared with previous reported approaches for iPSCs generation, mRNA–based methods are gaining progressively more attention due to the safety and high efficiency. mRNA induced iPSCs get avoid of the tumorigenic risks raised in retrovirus [1], lentivirus [19] and adenovirus [20] directed transfection and induction [21, 22].

In vitro preparation of mRNA from the four transcription factors is the key step in this approach. To initiate transcription in vitro, T7 or SP6 transcriptional promoters are required. pcDNA3.0 vector contains T7 promoter recognition regions and various internal restriction enzyme recognition sites, which is an optional tool to be used for T7 promoter induced in vitro transcription.

In our study, mRNA induced goat iPSCs from GEFs were similar as that of the human iPSCs obtained by Matthew [23] , and the morphological changes of the GEFs during the induction process were almost the same as that in non-mRNA induced human and mouse iPSCs [1, 24] and mRNA induced human iPSCs [25]. Our results illustrate that we successfully generated goat iPSCs with mRNA induction. The derived goat iPS cells share most of the characteristics as goat ES cell clones with respect to morphology, growth properties and expression of pluripotent markers. The results from western blot and qRT-PCR demonstrated the activation of endogenous Oct4, Sox2, Klf4, and c-Myc on day 12 after mRNA transfection, indicating the initiation of endogenous reprogramming mechanism at this time. Meanwhile, analysis of endogenous Nanog expression and the methylation status of its promoter further confirmed the activation of endogenous regulatory network on day 12 after mRNA transfection.

The first study regarding mRNA-induced reprogramming of iPSCs was reported by Warren et al. [8]. They used transcription vector carrying human Oct4, Sox2, Klf4 and c-Myc genes to obtain mRNA in vitro*,* and transfected the mRNAs into human fibroblasts to obtain human iPSCs. The feasibility of this approach was further validated by Heng et al. [26] using mRNA to reprogram human cells. In the process of mRNA induced animal iPSCs generation, the exogenous mRNA of oct4, sox2, klf4, and c-Myc transcription factor need to continuously function in the nucleus of transfected somatic cells for no less than 8 days, and maintain in a high expression level. Our data indicate that, after five-time continuous transfection, the mRNA in goat GEFs could be stably expressed for almost 9 days in the nucleus with a high level. Meanwhile, suppressing immunologic responses in target cells is another point we need to pay attention to. Matthew et al. [22] used siRNA to inhibit the expression of key proteins responsible for immunologic responses that induced by prolonged mRNA transfection, and they achieved somatic cells reprogramming by multiple transfection of Oct4, Sox2, Klf4 and Utf1 mRNAs.

## Conclusions

We successfully generated goat iPSCs by transfection of Oct4, Sox2, Klf4 and c-Myc mRNAs into GEFs, which initiated the endogenous reprogramming network and altered the methylation status of pluripotent genes. Core promoter regions and functional transcription binding sites of the four reprogramming genes were identified. Epigenetic regulation was revealed to participate in mRNA induced iPSCs formation. Our study provides a safe and efficient approach for goat. iPSCs generation.

## Additional file

Additional file 1: Table S1. The primer sequences for qRT-PCR. (DOCX 19 kb)

## Abbreviations

5-Azadc: 5-aza-2: deoxycytidine
EBs: Embryoid bodies
ESCs: Embryonic stem cells
FBS: Fetal bovine serum
GEFs: Goat embryonic fibroblasts
ICM: Inner cell mass
iPSCs: Induced pluripotent stem cells
TFs: Transcription factors
TSA: Trichostatin A
VPA: Valproic acid
