# Supplementary material for: Inducing goat pluripotent stem cells with four transcription factor mRNAs that activate endogenous promoters
Source: BMC Biotechnol. 2017 Feb 13;17:11. doi: 10.1186/s12896-017-0336-7 (PMC5307868; doi:10.1186/s12896-017-0336-7)
Supplement: Additional file 1: Table S1. — The primer sequences for qRT-PCR. (DOCX 19 kb) [file 12896_2017_336_MOESM1_ESM.docx]

| gene | Primer sequences |
| --- | --- |
| 4-Oct | F：5'GGCCAGAGCCGGGCTGGGTTGA3' |
|  | R：5'CGGGCTGAGGGGTCTC3' |
| Sox2 | F：5'GTGTGGTCCCGCGGGC3' |
|  | R：5'CCTTCATGTGCAGCGCTCG3' |
| Klf4 | F：5'ACTTCCCCCGGTGCTTC3' |
|  | R：5'GGCGGCCACGGACTC3' |
| c-myc | F：5'ACGAGGAGGAGAACT3' |
|  | R：5'TCCAGCTGGTCGGCC3' |
| Nanog | F：5'CAGAAAACGCTCCTGCC3' |
|  | R：5'TAGAAGCCCGGGTA3' |
| TERT | F：5'CGGAGACCACGTTCCAGAAG3' |
|  | R：5'GACAGTTCTCGAAGCCGCAC3' |
| Dax1 | F：5'GCCATGCCCCTCAACGTCAGTCGCC 3' |
|  | R：5'TTAGCAGCGCAAGAGTTCCGTATC 3' |
| AFP | F：5'TGGCGCAGGACACCTCGCTTCTGACT 3' |
|  | R：5'CGGCGAGTCAGTTATACGACCTAGA 3' |
| DCN | F：5'CACGACACGGACACAACCGACGCT 3' |
|  | R：5'TCCATGGTTTGCATTGGAGAACCA 3' |
| Neurod | F：5'TGGCTACGCAACGGGACACCATTG 3' |
|  | R：5'AAGTCTATGCATGCATAGGCCCAGA 3' |
| NFH | F：5'TGTTGGGTCGATTACGGTACTGTGCG 3' |
|  | R：5'TCGGCTTGTCGTAGGGTACCGATAC 3' |
| Myf5 | F：5'GTGAGCAACGTAGGACTGACG 3' |
|  | R：5'TGACTGTTTGACCCAGTGACCAGA 3' |
| Renin | F：5'TGATGCACCCGATGACAATGGGAA 3' |
|  | R：5'GCTGAAATGCTATACGTACAGAA 3' |
| Dnmt3b | F：5'GGCTATGCCGGGTTACGGGACCAC 3' |
|  | R：5'GGCGTAAAGTTGACCAGTGTAGGAC 3' |
| Gdf3 | F：5'GTAGACAGCAGGAGTGACCCAAGGA 3' |
|  | R：5'TGGATTTGGGTCGATCTGTGCGCGTTG 3' |
| TET1 | F：5'GAAGCCATCCGTCATTGTGTCTTTGGA 3' |
|  | R：5'AAAGTATGACAGGTATTGAAGTGG 3' |
| TET2 | F：5'CCAGACAAAGGCAGCAGTCAA 3' |
|  | R：5'CTTCTCAAAAACAAGGCCGTGCTC 3' |
| TET3 | F：5'CCCAACTGCAACTGCGATGGCCC 3' |
|  | R：5'CCAGATGCCTCCTCTGGAGG 3' |
| GAPDH | F：5'ATACTGGCAAAGTGGACATC3' |
|  | R：5'ATCACCCCACTTGATGT3' |

Table S1. The primer sequences for qRT-PCR
